# *Padina pavonica* Extract Promotes In Vitro Differentiation and Functionality of Human Primary Osteoblasts

**DOI:** 10.3390/md17080473

**Published:** 2019-08-15

**Authors:** Mariagiulia Minetti, Giulia Bernardini, Manuele Biazzo, Gilles Gutierrez, Michela Geminiani, Teresa Petrucci, Annalisa Santucci

**Affiliations:** 1 Dipartimento di Biotecnologie, Chimica e Farmacia (Dipartimento di Eccellenza 2018-2022), Università degli Studi di Siena, via Aldo Moro 2, 53100 Siena, Italy; 2 Institute of Cellular Pharmacology (ICP Ltd.), F24, Triq Valletta, Mosta Technopark, Mosta MST 3000, Malta

**Keywords:** *Padina pavonica*, marine algae, osteoporosis, bone metabolism, bone health, nutraceutical

## Abstract

Marine algae have gained much importance in the development of nutraceutical products due to their high content of bioactive compounds. In this work, we investigated the activity of *Padina pavonica* with the aim to demonstrate the pro-osteogenic ability of its extract on human primary osteoblast (HOb). Our data indicated that the acetonic extract of *P. pavonica* (EPP) is a safe product as it did not show any effect on osteoblast viability. At the same time, EPP showed to possess a beneficial effect on HOb functionality, triggering their differentiation and mineralization abilities. In particular EPP enhanced the expression of the earlier differentiation stage markers: a 5.4-fold increase in collagen type I alpha 1 chain (COL1A1), and a 2.3-fold increase in alkaline phosphatase (ALPL), as well as those involved in the late differentiation stage: a 3.7-fold increase in osteocalcin (BGLAP) expression and a 2.8-fold in osteoprotegerin (TNFRSF11B). These findings were corroborated by the enhancement in ALPL enzymatic activity (1.7-fold increase) and by the reduction of receptor activator of nuclear factor-*κ*B ligand (RANKL) and osteoprotegerin (OPG) ratio (0.6-fold decrease). Moreover, EPP demonstrated the capacity to enhance the bone nodules formation by 3.2-fold in 4 weeks treated HOb. Therefore, EPP showed a significant capability of promoting osteoblast phenotype. Given its positive effect on bone homeostasis, EPP could be used as a useful nutraceutical product that, in addition to a healthy lifestyle and diet, can be able to contrast and prevent bone diseases, especially those connected with ageing, such as osteoporosis (OP).

## 1. Introduction

Bone is a specialized form of connective tissue and its functions include locomotion, protection and mineral homeostasis. Osteoblasts, osteocytes, and bone lining cells are bone-forming cells, whereas osteoclasts are involved in the bone resorption process. The retention of homeostasis is based on the balance between these opposite activities. Therefore, bone is a very dynamic tissue due to the continuous balance between mineralization and resorption processes, that guarantee tissue homeostasis and functions [1]. With ageing, a net loss of bone is observed due to the increment of the resorptive activity of osteoclasts that is not balanced by novel bone tissue formation. This condition leads to pathological processes, such as osteoporosis (OP), a devastating bone disease [2] characterized by thinning of the tissue, changes in skeletal architecture, and significant increase of fracture risks. OP affects both women and men (even if it is more frequently observed in postmenopausal women), and the healthy and socio-economic issues connected to this pathology are expected to grow due to the increase of life expectancy. Due to the inefficiencies of current treatment options and related side effects, alternative therapies and preventive agents are highly desirable [3]. Osteogenic bioactive compounds have been isolated from many marine organisms, mainly macroalgae, such as brown algae *Sargassum horneri* and *Undaria pinnatifida*, so that OP could benefit from a novel and more efficient marine-based treatment. Compounds from marine organism are known to have a wide range of osteogenic effects, including stimulation of osteoblast functions and mineralization, as well as suppression of osteoclast activity [3,4].

Many previous studies have focused on the beneficial protective effects of seaweeds on human health and against chronic disease as they represent a source of unique bioactive compounds, such as proteins, peptides and amino acids, lipids and fatty acids, sterols, polysaccharides, oligosaccharides, phenolic compounds, photosynthetic pigments, vitamins, and minerals [5,6].

For the extraction of all the above-mentioned compounds, many different methods and solvents have been used. The process parameters of each method and the solvent must be chosen and optimized in order to obtain the extracts with the targeted bioactive compounds [7]. Parameters such as techniques, solvent, temperature, and raw material are known to notably affect the yield of extracted compounds from a quantitative and a qualitative point of view [6,8]. As the demand of macroalgae in the development of PUFA-related dietary supplements is growing, Kumari P. et al. performed a comparison of different lipid and fatty acid extraction and derivatization methods [9].

*P. pavonica* is a marine brown seaweed, a member of the Dictyotaceae family that is widespread throughout the world in warm temperate to tropical locations, including North Carolina to Florida in the United States, the Gulf of Mexico, throughout the Caribbean and tropical Atlantic and the Eastern Atlantic, Mediterranean, and Adriatic Seas [10]. In marine biology, *P. pavonica* is used above all as sensor or marker to study pollution levels in the sea and, in general, in the marine environment [11]. Regarding the functional and positive influence of *P. pavonica* on human health, sterols, lipids, polysaccharides, carotenoids, polyphenols, and fibers are the main bioactive compounds found in *Padina* species [12].

In a previous in vivo study conducted on 40 postmenopausal women and based on the initial founding by Gilles Gutierrez [13], *P. pavonica* demonstrated the ability to increase bone mineral density (BMD) and to exert a positive effect on collagen control (ICP Ltd., personal communication based on the study performed by Professor Mark Brincat). Nevertheless, based on our literature research, in vitro biochemical and molecular evaluation supporting osteogenic beneficial effects from *P. pavonica* extracts are nonexistent Therefore, in this study, we aimed to demonstrate the activity of EPP on bone homeostasis, providing the first report on French Polynesian *P. pavonica* effects on HOb metabolism. In particular, we undertook biochemical and molecular analyses to demonstrate if this vegetal substance may increase the uptake and the fixation of calcium by osteoblasts, and thus can induce a mass increment of bone tissue.

## 2. Results 

### 2.1. Chemical Composition and Antioxidant Capacity of EPP

EPP was chemically characterized for its total phenolic, flavonoid, and tannin content [6]. The total phenolic, flavonoid, and tannin contents of the seaweed were 27.0, 54.8, and 54.3 mg per g of extract, respectively, corresponding to 0.81, 1.64, and 1.63 mg per g of dry material, respectively. The antioxidant activity resulted as 256 ± 2 µmol of Fe^2+^ per g of extract.

**Figure 1:**
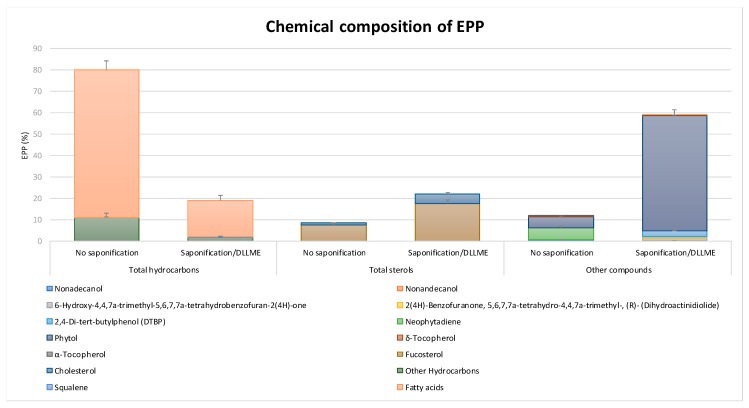
*Padina pavonica* extract (EPP) lipid content by GC-MS. For the analysis of the most lipophilic compounds, EPP was submitted to saponification and DLLME. Experiments were performed in triplicate. Data are presented as mean ± SD.

EPP was also examined for its lipid content by GC-MS [6]. Hydrocarbons represented 79.88% of the total extract, among which 68.83% corresponded to fatty acids (FAs), 0.19% corresponded to squalene, and 10.86% to other hydrocarbon species (Figure 1). Sterols represented 8.37% of the extract and included fucosterol and cholesterol at percentages of 7.40% and 0.97%, respectively (Figure 1). GC-MS analysis was also performed with a different sample preparation approach consisting in a saponification and an extraction by dispersive liquid-liquid microextraction (DLLME) of EPP, in order to analyze the most lipophilic compounds. This analysis mostly confirmed the presence of several already identified compounds (Figure 1).

**Figure 2:**
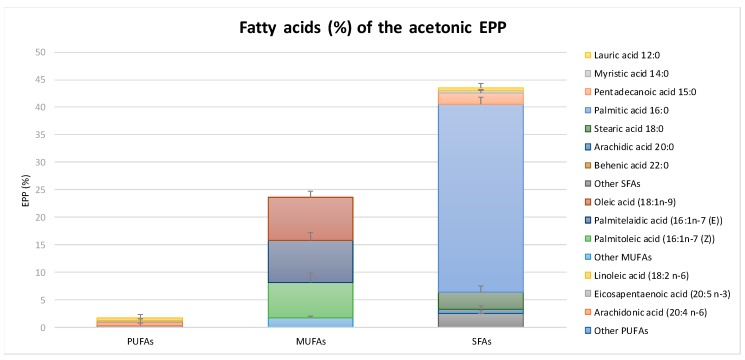
*Padina pavonica* extract (EPP) fatty acids (FAs) profile: PUFAs (polyunsaturated FAs), MUFAs (monounsaturated FAs) and SFAs (saturated FAs). Experiments were performed in triplicate. Data are presented as mean ± SD.

EPP’s FA profile (Figure 2) showed that the presence of saturated FAs (SFAs) corresponded to 43.45% of total EPP (63.13% of total FAs). Among these, the most abundant FA was palmitic acid with a total percentage of 34.15%, followed by stearic (3.25%), pentadecanoic (1.95%), arachidic (0.74%), myristic (0.43%), lauric (0.47%), and behenic (0.04%) acids. Monounsaturated FAs (MUFAs) made up 23.67% of total EPP (34.40% of total FAs). The most abundant MUFA was palmitelaidic acid (16:1 n-7 E, 7.82%), followed by oleic acid (18:1 n-9, 7.79%) and palmitoleic acid (16:1 n-7 Z, 6.29%). Polyunsaturated FAs (PUFAs) corresponded to 1.70% of EPP (2.47% of total FAs). The main PUFA found in EPP was arachidonic acid (20:4 n-6, 0.64%), followed by linoleic (18:2 n-6, 0.53) and eicosapentanoic acid (20:5 n-3 0.24%) [6].

### 2.2. EPP Effects on HOb Viability

**Figure 3:**
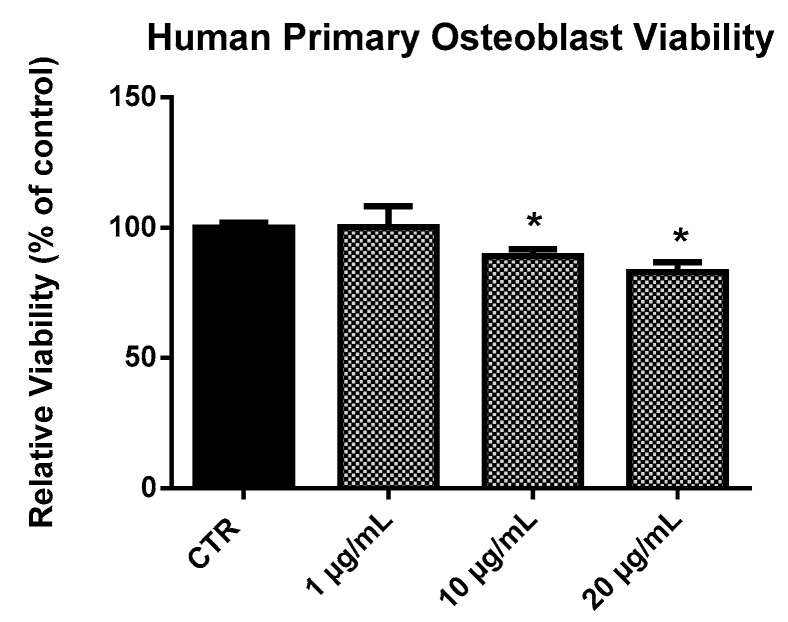
Viability of human primary osteoblasts (HOb) following 24 h treatment with EPP. Experiments were performed in triplicate. Data are expressed as percentage of control and presented as mean ± SD. Statistically significant differences from untreated control are denoted by * *p* < 0.05.

EPP did not exhibit significant effects on HOb viability at the concentrations used (1, 10, 20 µg/mL) after 24 h treatment (Figure 3). We detected a minor effect on HOb viability only at the highest concentrations tested. Therefore, having verified that EPP had no remarkable toxic effects at the concentrations tested, we focused on analyzing its functional activity on HOb.

### 2.3. Expression Analysis of Bone Differentiation Markers

To evaluate the effect of EPP at the molecular level, we extracted RNAs from EPP-treated cells and performed RT-qPCR analysis of osteoblastic-specific genes. Expression of genes coding for osteocalcin (BGLAP), osteoprotegerin (TNFRSF11B), collagen type I alpha 1 chain (COL1A1), alkaline phosphatase (ALPL) and Sox9 after 24 h of treatment with EPP at different concentrations (1, 10, and 20 µg/mL) was assessed. These mRNA species were chosen for our study as they represent recognized markers of the different osteoblast differentiation stages. Sox9 mRNA was also monitored as a transcription factor involved in cartilage growth during chondrogenesis. At the molecular level, Sox9 directly interacts with RUNX2, a transcription activator of osteoblast-specific genes, decreasing RUNX2 binding to its target sequences and inhibiting its activity. During osteochondroprogenitor cells’ differentiation toward the osteoblastic phenotype, Sox9 expression levels decrease and RUNX2 increases [14].

#### 2.3.1. *BGLAP*

**Figure 4:**
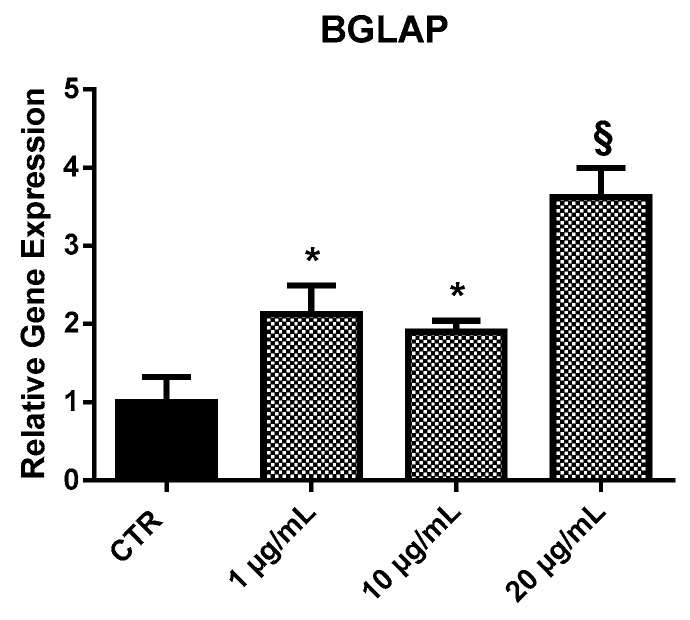
Relative expression of the *BGLAP* gene from HOb following 24 h treatment with EPP. Untreated cells were used as controls. Experiments were performed in triplicate. Results are shown as a mean of fold change in gene expression ± SD using untreated osteoblasts as a control. Statistically significant differences from untreated control are denoted by * *p* < 0.05 or § *p* < 0.01.

EPP treatment induced an increment of *BGLAP* expression in HOb at all the concentrations tested (Figure 4). In particular, a nearly two-fold increase of *BGLAP* was observed at 1 and 10 µg/mL, and of around 3.7-fold at 20 µg/mL.

#### 2.3.2. *TNFRSF11B*

**Figure 5:**
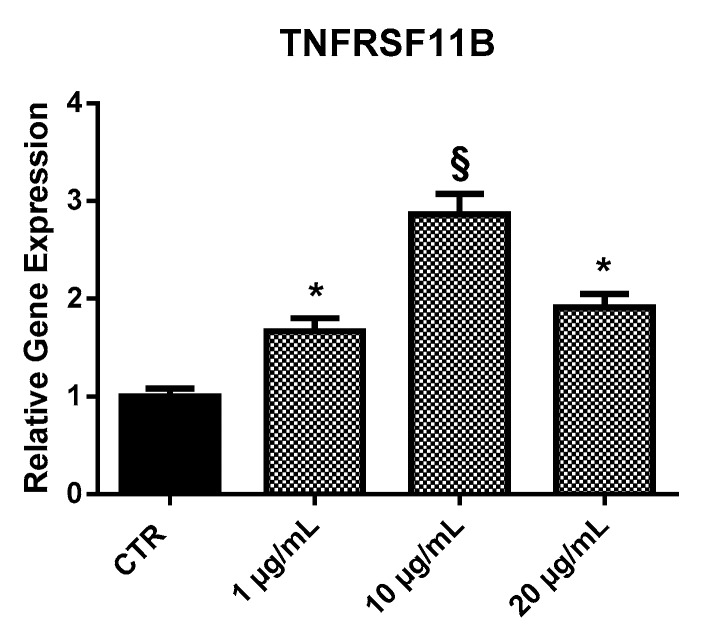
Relative expression of the *TNFRSF11B* gene from HOb following 24 h treatment with EPP. Experiments were performed in triplicate. Results are shown as a mean of fold change in gene expression ±SD using untreated osteoblasts as a control. Statistically significant differences from untreated control are denoted by * *p* < 0.05 or § *p* < 0.01.

EPP treatment induced an increase of *TNFRSF11B* expression in HOb. In particular, all the concentrations tested induced statistically significant increase of the gene expression: 1.7-fold at 1 µg/mL, 2.8-fold at 10 µg/mL, and nearly two-fold at 20 µg/mL (Figure 5).

#### 2.3.3. *COL1A1*

**Figure 6:**
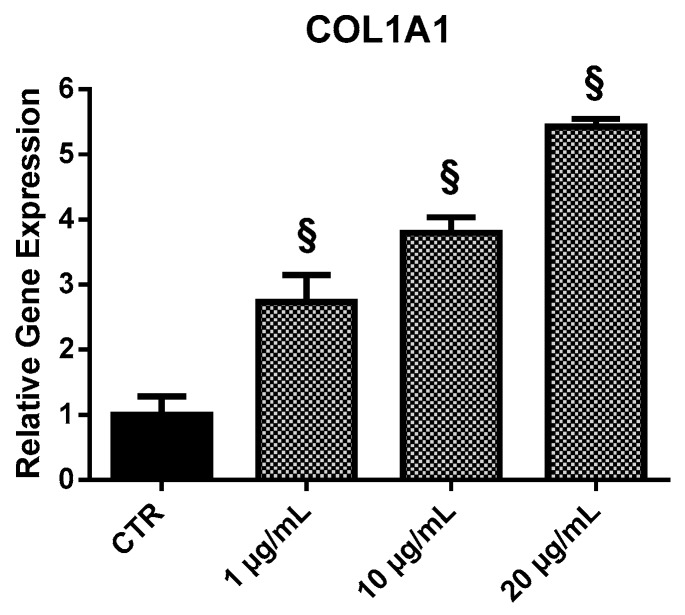
Relative expression of the *COL1A1* gene from HOb following 24 h treatment with EPP. Experiments were performed in triplicate. Results are shown as a mean of fold change in gene expression ± SD using untreated osteoblasts as a control. Statistically significant differences from untreated control are denoted by § *p* < 0.01.

*COL1A1* was expressed in larger amounts in EPP-treated HOb in respect to control (Figure 6). EPP treatment induced a dose-dependent increase in *COL1A1* expression: 2.7-fold at 1 µg/mL, 3.8 at 10 µg/mL, and 5.4-fold at 20 µg/mL.

#### 2.3.4. *ALPL*

**Figure 7:**
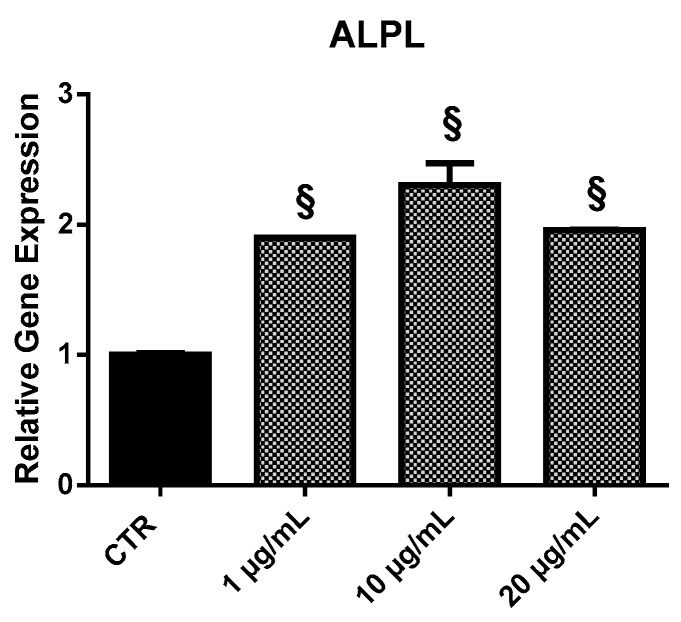
Relative expression of the *ALPL* gene from HOb following 24 h treatment with EPP. Experiments were performed in triplicate. Results are shown as a mean of fold change in gene expression ± SD using untreated osteoblasts as a control. Statistically significant differences from untreated control are denoted by § *p* < 0.01.

HOb showed an increase expression of *ALPL* following EPP treatment (Figure 7). The expression of the gene was enhanced by nearly 1.9-fold at 1 µg/mL, 2.3-fold at 10 µg/mL, and nearly two-fold at 20 µg/mL.

#### 2.3.5. *Sox9*

**Figure 8:**
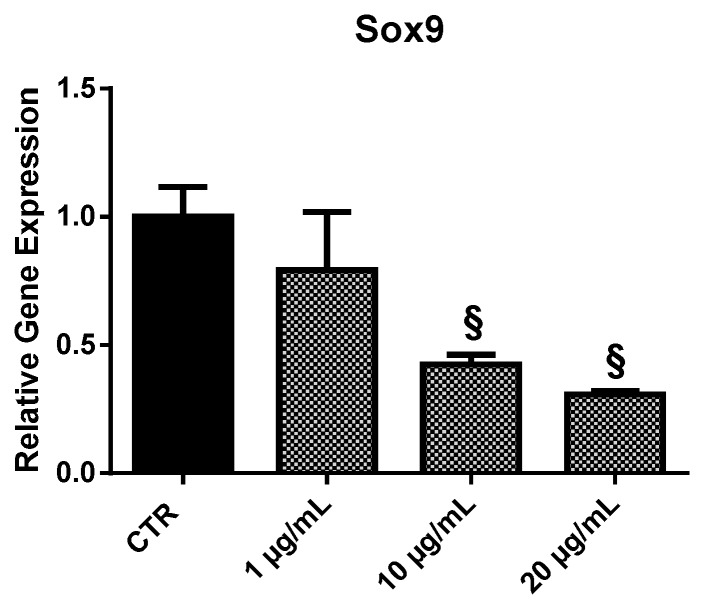
Relative expression of the *Sox9* gene from HOb following 24 h treatment with EPP. Experiments were performed in triplicate. Results are shown as a mean of fold change in gene expression ± SD using untreated osteoblasts as a control. Statistically significant differences from untreated control are denoted by § *p* < 0.01.

EPP treatment of HOb resulted in a dose-dependent reduction of *Sox9* expression compared to control (Figure 8): 0.8-fold at 1 µg/mL, 0.4-fold at 10 µg/mL, and 0.3-fold at 20 µg/mL.

### 2.4. Receptor Activator of Nuclear Factor-κB Ligand (RANKL) and Osteoprotegerin (OPG) Ratio (RANKL/OPG Ratio)

**Figure 9:**
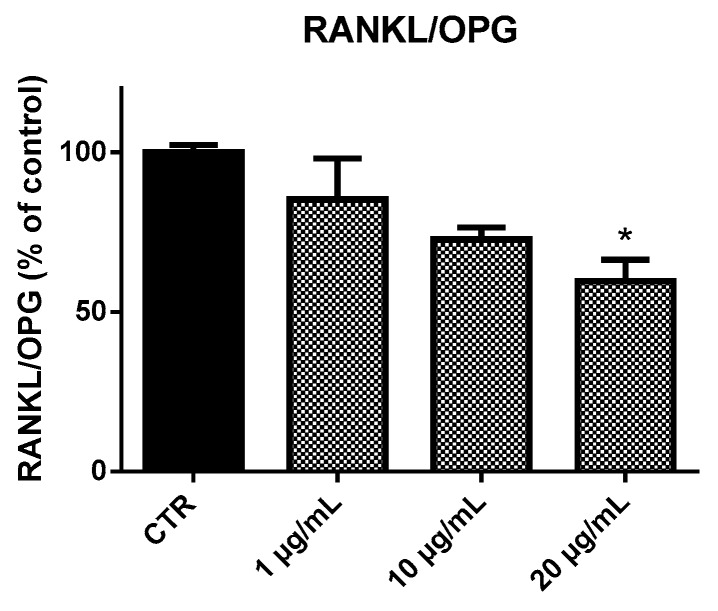
RANKL/OPG levels in HOb following 96 h treatment with EPP. Experiments were performed in triplicate. Data are expressed as percentage of control and presented as mean ± SD. Statistically significant differences from untreated control are denoted by * *p* < 0.05.

The RANKL/OPG ratio is the main determinant of bone mass and better reflects the bone remodeling condition [15]. In EPP-treated HOb, we detected a reduction in RANKL/OPG ratio with increasing EPP concentrations (Figure 9). In particular, EPP at 20 µg/mL caused a reduction in RANKL/OPG ratio of nearly 0.6-fold compared to untreated HOb (CTR).

### 2.5. Alkaline Phosphatase (ALPL) Activity

**Figure 10:**
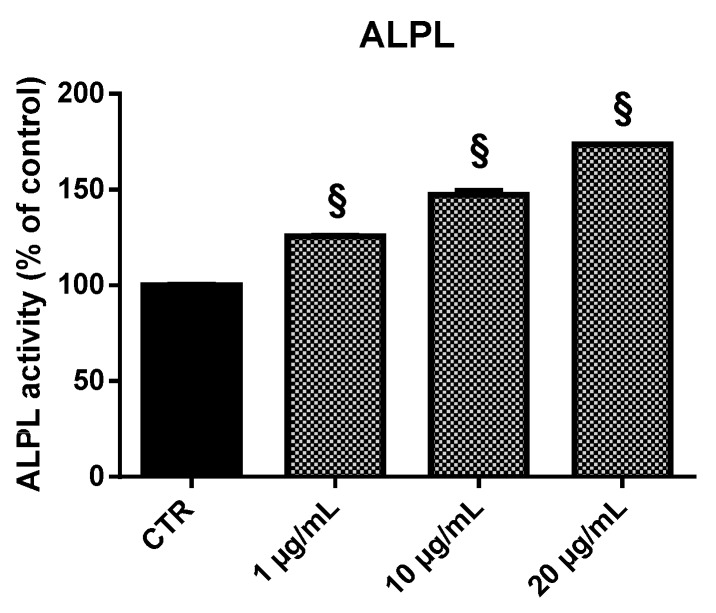
ALPL activity in HOb following 96 h treatment with EPP. Experiments were performed in triplicate. Data are expressed as percentage of control and presented as mean ± SD. Statistically significant differences from untreated control are denoted by § *p* < 0.01.

ALPL activity was detected following 96 h treatment with EPP. Compared with control cell cultures, EPP treatment significantly upregulated ALPL activity in HOb (Figure 10). In particular, EPP treatment at 1, 10, and 20 µg/mL led to an increase in ALPL enzymatic activity of 1.25, 1.5, and 1.7-fold over the control, respectively.

### 2.6. Bone Nodule Formation and Mineralization

Detecting the formation of mineralized nodules in EPP-treated HOb cultures has provided a means to assess mature osteoblast cells’ function and the status of the cultures.

**Figure 11:**
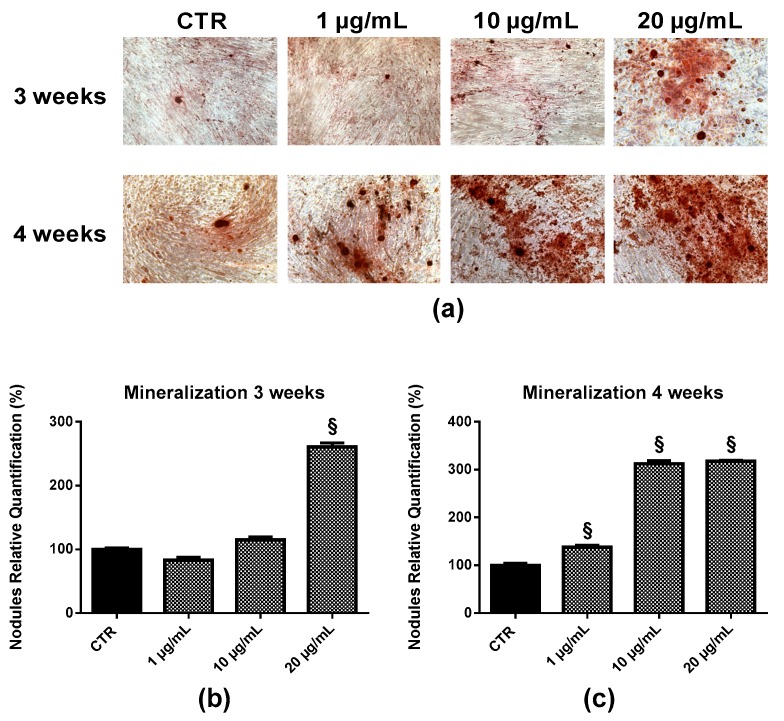
Detection (**a**) and quantification (**b**,**c**) of mineralized nodules formed in HOb cultured for 3 weeks (**b**) or 4 weeks (**c**) in the presence of EPP at different concentrations. Experiments were performed in triplicate. Bars represent mean ± SD. Original magnification: 10×.

EPP treatment of HOb for 3 or 4 weeks induced the deposition of mineralized nodules (Figure 11a). The nodules appeared three-dimensional under a phase contrast microscope and continued to grow until the end of the culture period.

Quantifying the mineralized nodules after 3 weeks indicated no significant difference in HOb treated with EPP at 1 and 10 µg/mL compared to the control, whereas 20 µg/mL EPP induced a 2.6-fold increase in calcium deposition (Figure 11b). At 4 weeks EPP treatment, a larger increase in calcium deposition was observed at all the concentrations of EPP tested: 1.4-fold at 1 µg/mL, 3.1-fold at 10 µg/mL, and 3.2-fold at 20 µg/mL (Figure 11c).

## 3. Discussion

OP is a silent disease which leads to feeble quality of life and increased mortality in aged people, especially in postmenopausal women [16]. The balance between bone resorption and bone formation is the key point in bone homeostasis and health and an imbalance of these events causes OP. Loss of bone matrix and mass and microarchitectural deterioration are the main features of OP that increase the rate of fractures [16]. Nowadays, finding the proper treatment for bone-related disease is a matter of great interest. Due to the inefficiencies of current treatment options and related side effects, alternative therapies and preventive agents are highly desirable [3]. Since natural products are showing lower side effects and are more suitable for long-term use, they are quickly replacing traditional synthetic drugs [16].

Osteogenic bioactive compounds have been isolated from many marine organisms, mainly macroalgae, such as brown algae *Sargassum horneri* and *Undaria pinnatifida*, so that OP could benefit from a novel and more efficient marine-based treatment. Compounds from marine organisms are known to have a wide range of osteogenic effects, including stimulation of osteoblast functions and mineralization, as well as suppression of osteoclast activity [3,4].

Marine algae have been demonstrated to be strong candidates for the extraction and enforcement of novel drugs [17] and in recent years, significant development has been achieved in the isolation of these active compounds with several activities, such as anticancer, anti-inflammation, antioxidant, and having an inhibitory effect on ROS generation [18].

Numerous macroalgae have shown potent cytotoxic activities and some authors have suggested the utilization of algae as a chemopreventive agent against several cancers. Among these, extracts from *Laurencia viridis* and *Portieria horemanii*, containing dehydro-thrsiferol and halomon, have been tested in preclinical trials [19,20]. Recently, the methanolic extract of *P. pavonica* from the Adriatic Sea (Montenegro) was demonstrated to possess antitumoral activities on human cervical and breast cancer cell lines [21], inducing high DNA damage and cell growth inhibition due to apoptosis. Moreover, we previously demonstrated the proapoptotic activity of French Polynesian *P. pavonica* extract on human osteosarcoma cells. These finding suggests that EPP could be of special interest for developing novel therapeutic agents for osteosarcoma, a rare highly malignant bone cancer, whose cells phenotypically present an early stage of differentiation [2].

Extract or bioactive compounds from macroalgae have been shown to possess a noticeable effect on regulation of bone metabolism as proved by enhanced bone mass, trabecular bone volume, number and thickness and lower trabecular separation, resulting in a higher bone strength [17]. Such anti-OP effects seem to be mediated via antioxidant or anti-inflammatory pathways and their downstream signaling mechanisms, leading to osteoblast mineralization and osteoclast lack of activity [17].

Seaweeds not only consist of organic bio-active compounds such as phenols, flavonoids and tannins, fatty acids, polysaccharides, proteins, and fibers, but they are also a valuable source of minerals such as calcium, magnesium, and other bone-supporting elements [22]. Mineral-rich extracts have been isolated from the red marine algae *Lithothamnion calcareum* and tested as a dietary supplement for prevention of bone mineral loss [22]. The extract of the brown algae *Sargassum horneri* has been demonstrated to possess an anabolic effect on bone elements, due to its capacity to stimulate bone deposition and inhibit bone degradation in rat femoral tissues in vitro and in vivo [23].

The effect of algae such as *Undaria pinnatifida*, *Sargassum horneri*, *Eisenia bicyclis*, *Cryptonemia scmitziana*, *Gelidium amasii*, and *Ulva pertusa Kjellman* on bone calcification have been studied. Results showed that bone calcium content was significantly increased [22,24].

The methanol extract of brown algae *Ecklonia cava* has been used for in vitro arthritis treatment [25].

Nevertheless, very few compounds have been analyzed and reported for bone-related disease treatment and the effect of marine algae extracts on bone metabolism has not yet been entirely clarified. Still much research work is needed for further elucidations.

In this work, we investigated the proanabolic activity of *P. pavonica* on HOb by monitoring the effects of EPP on cell viability, differentiation, and mineralization.

EPP was previously characterized for its chemical composition; in particular, we determined the total phenolic, flavonoid, and tannin content, antioxidant activity, lipid composition, and fatty acid profile [6].

Regarding the effect of the brown algae *P. pavonica* on bone metabolism, in a previous in vivo study, the activity of a marine algae-derived molecules on bone density and collagen synthesis markers were investigated. Briefly, 40 postmenopausal women were recruited and randomly treated with different dose of *P. pavonica.* Every 3 months, physical examination, including bone densitometry and collagen markers measurement, was conducted. At the end of the 12-month period, an ultrasound scan and cervical cytology analysis were conducted. *P. pavonica* demonstrated the ability to increase BMD measured in lumbar spine and femur neck compared to the untreated group. Regarding collagen analysis, procollagen I C-end terminal peptides and pyridinium crosslinks were investigated as markers of bone formation and bone resorption, respectively. Results revealed that *P. pavonica* may have a positive effect on collagen control. Finally, *P. pavonica* did not appear to affect other estrogen-sensitive organs such as the endometrium or vaginal mucosa. Steroid structure compounds were suggested as the active molecules responsible for the observed effects. Such results led to the hypothesis of a selective estrogen receptor modulator-like molecules (ICP Ltd., personal communication based on the study performed by Professor Mark Brincat).

Nevertheless, based on our literature research, in vitro biochemical and molecular evaluation supporting beneficial osteogenic effects of *P. pavonica* extracts are nonexistent. Hence, in this study, for the first time, the biological activity of EPP was evaluated on HOb.

Overall, our data indicate that EPP is a safe product regarding cell viability, showing no toxicity against HOb. RT-qPCR was used to examine the expression of ALPL, collagen type I alpha 1 chain, osteoprotegerin, and osteocalcin. These mRNA species were chosen for our study as they represent recognized markers of the different osteoblast differentiation stages. COL1A1 and ALPL characterized the earlier stage; in the late stage, matrix mineralization occurs when the organic structure is supplemented with osteocalcin, which stimulates deposition of mineral substances [26]. EPP exhibited the capacity to increase the expression of the earlier differentiation-stage markers (COL1A1 and ALPL) as well as those involved in terminally osteoblastic differentiation (BGLAP and TNFRSF11B). In accordance with these findings, EPP also showed the ability to increase ALPL enzymatic activity. Sox9 mRNA was also monitored. Sox9 is a chondrocyte-specific transcription factor and it is required for prechondrogenic cell condensation and prechondrocyte and chondroblast differentiation [27]. The SOX9 and RUNX2 expression ratio is crucial in determining the shift in equilibrium toward osteogenesis or chondrogenesis [28]. RUNX2 regulates downstream genes that determine the osteoblast phenotype and controls the expression of osteogenic marker genes such as ALPL, Osteopontin (OPN), Osterix (OSX), COL1A1, Bone sialoprotein (BSP), and BGLAP [28].

Zhou G. et al. [14] in their study identified a transcriptional repressor function of Sox9 on RUNX2 acting during chondrogenic cell fate commitment and chondrogenesis. There are evidences on the dominance of Sox9 function over RUNX2 during the early first step in the progenitor cell fate decision between osteoblastic vs. chondrogenic lineages. It has been shown that Sox9 misexpression repressed RUNX2 function and diverted cell fate from bone to cartilage in the craniofacial region [14]. Based on these evidences, we selected Sox9 as a marker for the osteoblast phenotype maintenance in order to prevent osteoblasts from shifting toward the chondrogenic lineage. Regarding EPP treatment, a decrease in Sox9 expression was detected in treated osteoblast culture compared to untreated culture.

RANK, RANKL, and OPG have a fundamental role in bone remodeling and the RANKL/OPG ratio is the main determinant of bone mass and better reflects the bone remodeling condition [29]. Our results showed that EPP upregulated OPG expression in HOb compared to the control culture. OPG acts as nonfunctional receptor to compete with the osteoclast activation receptor RANK for its ligand RANKL. Therefore, EPP showed an indirect inhibition effect on osteoclast activation. Finally, results showed a decrement in RANKL/OPG ratio as a demonstration of EPP capacity to inhibit bone resorption.

In the final step, the mineralizing ability of EPP was evaluated by Ca^2+^ deposition assay through an Alizarin red S (ARS) staining assay. EPP was found to significantly enhance mineralized nodule formation in osteoblast cultures. After 3 or 4 weeks, considering the tested concentrations of EPP (1, 10, 20 µg/mL), the extract did not have toxic effects on the cultures as cells were still vital, visibly attached, and occupying the entire bottom of the plate as compared with those of the control group. Mineralized nodules were observed in cells cultured in the absence of common mineralization agents such as dexamethasone and betaglycerophosphate, demonstrating the remarkably ability of the EPP to induce mineralization and indicating that this product serves as a suitable mineralized-nodule-inducing factor. Calcium level is crucial in the strengthening of bone and bone homeostasis. Regarding the therapeutic potential of marine algae in calcium-mineralization of osteoblasts, some phlorotannins have been identified as bioactive components in *Ecklonia* sp. [30].

Since there have been no previous reports, to our knowledge, this work can be considered the first to demonstrate the osteogenic capacity of *P. pavonica* extract in vitro. In the present study, we have shown EPP to be able to increase the deposition of mineralized organic matrix by osteoblasts through an increase of osteoblastic differentiation. The present study is the first to investigate the direct effects of EPP on bone-forming osteoblasts, providing evidences both at the molecular and cellular level. We demonstrated that EPP has a strong modulatory effect on the expression of osteoblast-specific markers such as: COL1A1, ALPL, BGLAP, TNFRSF11B genes, ALPL enzymatic activity, as well as on the RANKL/OPG ratio and on formation of mineralized bone nodules in long-term HOb cultures. This is important with regard to developing materials for bone repair or bone tissue engineering/regeneration, or active nutritional supplements.

## 4. Materials and Methods

### 4.1. Chemical Composition of EPP

EPP was produced and chemically characterized as previously described [6]. Briefly, EPP was produced by Soxhlet extraction using acetone as the solvent, starting from algae collected in French Polynesia in June 2014. EPP was first tested for its total phenolic, flavonoid, and tannin content through spectrophotometric assay (Folin–Ciocalteu method, aluminum chloride colorimetric method, Broadhurst vanillin–HCl method, respectively) [6]. The determination of the antioxidant activity of the extract was performed using the method of FRAP assay [6]. Finally, EPP was examined for its lipid content by GC-MS. For the analysis of the most lipophilic compounds, EPP was subjected to saponification and dispersive liquid-liquid microextraction (DLLME) [6].

### 4.2. Isolation and Culture of HOb 

HOb were isolated from the trabecular bone of adult knee samples obtained with ethical approval and informed consent during routine replacement surgery. Trabecular bone fragments were widely washed in PBS pH 7.4 to remove blood and bone marrow, and then transferred to culture containing DMEM (PAN Biotech) supplemented with 10% *v*/*v* fetal bovine serum (FBS) (Ultra-low endotoxin, Euroclone), and 1% *v*/*v* penicillin–streptomycin. Cultures were incubated at 37 °C in a humidified atmosphere of 5% CO_2_. Bone fragments were maintained in culture by removing the conditioned medium and replacing it with a fresh one, every 2 weeks. After 3–6 weeks in culture, a cellular confluent monolayer of Hob had grown out from the bone fragments (E1 culture) [31].

### 4.3. Cell Culture and Treatment

HOb were seeded at a density of 3000 cells/well into a 96-well multiplate for MTT assay, or at a density of 15,000 cells/well into a 24-well multiplate for ARS assay or total RNA extraction, and cultured in DMEM supplemented with 10% *v*/*v* FCS and 1% *v*/*v* penicillin–streptomycin. Subconfluent cells were treated with EPP obtained as previously described [6] at 1 µg/mL, 10 µg/mL, and 20 µg/mL and using DMSO as control, for 24 h for MTT assay or RNA extraction and for 96 h for RANKL and OPG ELISA kit. Alternatively, for ARS assay, confluent cells were treated at the same EPP concentrations for 3 or 4 weeks; fresh medium (containing EPP or DMSO) was replaced twice a week.

### 4.4. MTT Assay

Cell viability was determined after 24 h of treatment. Culture medium was removed and cells were incubated with MTT in white DMEM for 3.5 h. After incubation time, Formazan salts were dissolved in DMSO and absorbance was evaluated by a microplate reader with at 550 nm.

### 4.5. RT-qPCR

Each cultured construct was independently collected after 24 h of treatment. Total RNA extraction and cDNA synthesis were obtained using the FastLane Cell cDNA kit (Qiagen, Milano, Italy) with a TProfessional Basic Thermocycler (Biometra, Cinisello Balsamo-Milano, Italy), following manufacturer’s instruction. The RT-qPCR analyses were then performed with a RotorGene 6000 (Qiagen, Milano, Italy) using the SYBR® GreenERTM qPCR SuperMix Universal kit (Invitrogen Thermo Fisher, Monza, Italy). Target genes were amplified using specific primer pairs obtained from KiCqStart™ Primers (Sigma Aldrich, Milano, Italy). For each sample, the quality of the PCR product was tested by melting curve analysis. The results were expressed as fold change (increase or decrease) in expression of the treated sample in relation to the untreated sample. Glyceraldehyde-3-phosphate dehydrogenase (GAPDH) was used as reference gene to control for experimental variability and the level of mRNA expression was normalized to GAPDH mRNA. RT-qPCR analysis was performed in duplicate on samples taken from three independent cultures (i.e., six measurements for each gene).

### 4.6. ALPL Assay

After 96 h treatment, ALPL activity was quantified following the method described by Lowry and modified by Tsai [32]. Briefly, cells were washed with PBS and cells were lysed with 100 μL of 0.1% SDS. A total of 100 μL of the lysate was incubated with 250 μL p-nitrophenyl phosphate in glycine buffer at 37 °C for 30 min. The enzymatic reaction was stopped by adding 100 μL of ice-cold 3 M NaOH, and the amount of p-nitrophenol liberated was measured spectrophotometrically (405 nm). Each experiment was performed in triplicate and results were normalized to cell protein content.

### 4.7. Quantitative Detection of RANKL and OPG

OPG and RANKL release into the culture medium were measured after 96 h of treatment using their respective ELISA kit (Abcam, Cambridge, UK) following the manufacturer’s instructions. Optical density was read at 450 nm wavelength.

### 4.8. Nodules Formation and Mineralization Assay

Mineralized nodules formation and degree of mineralization were determined in HOb cells treated with EPP at 1 µg/mL, 10 µg/mL, and 20 µg/mL and using DMSO as control, for 3 or 4 weeks, fresh medium (containing EPP or DMSO) was replaced twice a week. After 3 or 4 weeks treatment, cells were submitted to ARS staining as described [33]. Briefly, cells were fixed with 70% *v*/*v* cold ethanol for 1 h and stained with 40 mM ARS stain in dH_2_O (pH 4.1) at RT for 20 min. Cells were washed five times with dH_2_O and two times with cold PBS. Mineralized ARS-positive nodules present in each well were visualized using inverted microscope. For the quantification of mineralization, ARS was extracted with 10% cetylpyridinium chloride (CPC) in PBS for 1 h, followed by absorbance measurement at 550 nm.

### 4.9. Statistical Analysis

Experiments were performed in triplicate. Data were expressed as mean ± SD. Statistical significance of differences was determined by ANOVA analysis, with a Bonferroni post hoc test. Statistically significant differences from untreated control are denoted by * *p* < 0.05 or § *p* < 0.01. Differences were considered significant at *p* < 0.05 (Graphpad; San Diego, CA, USA).
